# Law and Stock Market Development in the UK over Time: An Uneasy Match

**DOI:** 10.1093/ojls/gqad019

**Published:** 2023-08-11

**Authors:** Brian R Cheffins, Bobby V Reddy

**Affiliations:** SJ Berwin Professor of Corporate Law, Faculty of Law, University of Cambridge

**Keywords:** stock markets, stock exchange regulation, company law, nationalisation, privatisation

## Abstract

Britain has a reputation for having a stock market-oriented corporate economy and there is an extensive literature maintaining that laws affording substantial protection to outside investors are needed for a thriving stock market. Historically, however, UK equity markets have not always flourished and, when they have, law’s contribution has been open to question. This article considers the uneasy match between law and Britain’s stock market development from when shares first began to trade publicly through to the present day, offering in so doing insights into the relationship between law and equity markets and current reforms intended to revive a flagging UK stock exchange.

## 1. Introduction

In 2021, Rishi Sunak, then Chancellor of the Exchequer, declared, in relation to the stock market, that ‘Strong public markets are a vital component of the UK economy’.^1^ This accords with academic assessments of the stock market’s status in Britain. The UK has been characterised as a stock market citadel,^2^ having, the United States aside, a uniquely well-developed equity market ‘both historically and today’.^3^ Britain’s stock market origins are traceable back to the 16th century.^4^

While Sunak, as Chancellor of the Exchequer, indicated that a robust equity market is an important part of the British economy, recent trends are discouraging. A steady decline in the number of publicly traded companies in the UK has elicited fears that Britain’s stock market is ‘fading away’.^5^ Such concerns prompted Sunak in 2020 to commission, as part of an effort to fortify London’s position as a global financial centre, a review of regulatory arrangements of companies seeking to list their shares for trading on the London Stock Exchange (LSE).^6^ The resulting 2021 report made various proposals to relax or simplify existing requirements, emphasising, in so doing, that ‘The UK needs strong public markets’.^7^ The Financial Conduct Authority (FCA), which is responsible for promulgating and enforcing the Listing Rules to which companies listed on the LSE must adhere, implemented many of the recommendations.^8^ In 2023, the FCA issued a fresh set of proposals designed to make the UK listing regime ‘more straightforward’, indicating it wanted ‘to make sure that the UK public markets remain an attractive and trusted place to list companies to support growth and innovation’.^9^

Given Britain’s reputation as a stock market-oriented corporate economy and given the lengthy history of the stock market in Britain, it might be thought that the current weakness of equity markets is an aberration. Moreover, the fact that doubts about the strength of the stock market have elicited reforms intended to correct matters seems entirely logical. There is an extensive literature maintaining that the emergence of well-developed equity markets is contingent upon suitable laws being in place that afford substantial protection to outside investors.^10^ Moreover, a policy-making presumption in the UK that the stock market is an economic asset worthy of fostering and protecting would seem to have been a fully justified one, given that various media commentators,^11^ academics^12^ and policy makers^13^ maintain that a thriving stock market helps to foster a vibrant economy.

The position with respect to the strength of UK equity markets and law’s role in fostering stock market development is more complicated than the foregoing tidy law and stock market logic implies. While the government is currently seeking to use regulatory change to bolster the stock market, to the extent equity markets have thrived over the centuries in Britain, it is doubtful whether law’s contribution has been crucial. Contrary to the conventional wisdom that robust investor protection is a precondition for a well-developed stock market, until well into the 20th century the default regulatory stance in the UK was ‘hands-off’. More recently, in the early 2010s, stock market misgivings may well have precluded reforms explicitly intended to reverse a decline for which there was growing evidence.

In addition, public policy in the UK has periodically been antithetical to stock market development. Such regulation can be traced back to the early 18th century, and governmental policies contributed to a substantial, if largely forgotten, stock market reversal during the middle decades of the 20th century.^14^ Ultimately, then, while numerous commentators maintain strong investor protection is essential for stock market development, given the default ‘hands-off’ policy-making stance and given various reforms antithetical to stock market development, equity markets have historically flourished in the UK despite regulation more than because of it. The current era stands out as an aberration both because regulatory reform currently underway is designed explicitly to bolster the stock market and because the emphasis is on deregulation rather than bolstering investor protection.

There is an extensive literature canvassing the history of British equity markets which is continuing to expand.^15^ Substantial work has also been done on law’s contribution to stock market development in the UK.^16^ Little emphasis has been placed, however, on the notion that there has been an uneasy match between stock market development and public policy in Britain. More precisely, a point this article makes that has not been developed previously in any detail in the growing literature on British equity markets is that the stock market has thrived historically in the UK as much in spite of as because of stances law makers have adopted. This article is well situated to make this novel assertion because it offers a broader historical sweep than is standard in the existing literature and takes into account recent debates concerning the state of the stock market in the UK that throw past patterns into sharp relief.

We begin by providing context in the form of a succinct overview of the thesis that suitable investor protection is a precondition for a well-developed stock market. The article then addresses UK developments chronologically, starting with nascent 17th-century equity markets. The Bubble Act of 1720,^17^ a strikingly anti-stock market 18th-century reform, is canvassed next. We will then see that some commentators argue that the laissez-faire approach to company law during the second half of the 19th century and the early 20th century impaired domestic equity markets and thereby contributed to Britain’s industrial ‘decline’ relative to leading national rivals. That charge is dubious, partly because in various ways the stock market was well developed during this era. Nevertheless, lax company law featured generally until the mid-20th century, an era when government policy dealt various blows to equity markets.

While government policy contributed to a mid-20th-century stock market ‘dark age’, the tables turned in the 1980s, when various regulatory choices helped to fortify a stock market ‘golden era’. However, policy-maker enthusiasm began to ebb in the 1990s, and was slow to revive despite growing evidence of stock market decline. As we point out in the final substantive section of our article, the tide only turned fully in 2020, when UK policy makers began to treat bolstering the stock market as an explicit priority as it became clear that Britain’s status as a stock market citadel was in serious jeopardy.

## 2. Law and Stock Market Development

Law professor Bernard Black maintained in a 2000 article that it is ‘almost magical’ that strong securities markets exist at all, and suggested that ‘This magic does not appear in unregulated markets’.^18^ A ‘law and finance’ literature oriented around quantitative comparative analysis of the relationship between national legal institutions on the one hand and financial systems on the other has done much to lend credence to such reasoning.^19^ A key precept of this literature is that, due to information asymmetries, potential managerial agency costs and possible extraction of private benefits of control, there is a real danger that corporate ‘insiders’ (controlling shareholders and senior executives) will cheat outside investors who own equity. Given this danger, well-developed corporate and securities law does much to explain the existence of strong equity markets.^20^

To elaborate, the ‘law matters’ account assumes minority shareholders will feel ‘comfortable’ in a ‘protective’ corporate law environment.^21^ Such confidence means that investors will be willing to pay full value for shares made available for sale, which, in turn, will lower the cost of capital for firms that choose to sell equity in financial markets. Public offerings of shares can then easily follow. Laws identified as important in this context have included those dealing with voting rights and precluding better informed insiders from fleecing outside investors, such as by making remedies available to minority shareholders subjected to oppressive conduct and by mandating substantial corporate disclosure, restricting insider dealing of company shares and regulating potentially one-sided related party transactions.^22^

The theory that ‘Strong legal protection for shareholders appears to be a necessary condition for diffuse equity investment’^23^ has been highly influential.^24^ Doubts have been cast, however, on the accuracy of the coding of laws relied upon in empirical tests establishing a statistical link between laws protecting investors and robust securities markets.^25^ It is also not entirely clear which laws are crucial for stock market development.^26^ For instance, financial economists who generated and deployed in the late 1990s a highly influential anti-director rights index (ADRI) focusing on six aspects of company law subsequently concluded that less well-known indices of theirs focusing on mandatory disclosure associated with the public issuance of shares and regulation of related party transactions were superior measures of shareholder protection.^27^

History is also ‘a fly in the ointment of the law and finance hypothesis’.^28^ If law truly determines whether equity markets will flourish, the stock market should languish in a particular country at least until the law protects investors well. Various commentators maintain, however, that the UK had a well-developed stock market decades before company and securities law afforded investors substantial assistance,^29^ and the same point has been made in relation to Brazil and the United States.^30^

Using history to cast doubt on ‘law matters’ reasoning leads to an obvious follow-on question: if corporate and securities law is doing little to address concerns that investors will reasonably have about owning shares in publicly traded companies, how will equity markets flourish?^31^ There are various factors that can operate as potential substitutes that underpin demand for shares. A number will be canvassed in subsequent sections. To anticipate, a stock exchange can put in place conditions for the listing of shares that afford protection to outside investors, companies can of their own volition offer features that will reassure investors (eg paying cash dividends annually can serve as a potentially reliable signal of financial health) and there can be periodic bursts of investor optimism concerning shares that obscure the risks involved.^32^

## 3. Origins (up to 1720)

The Russia Company, the first English ‘joint stock’ company, in the sense that its members (shareholders) traded via one account in which all members had a transferable stake rather than doing so separately, was established by royal charter in the mid-1550s to exploit a monopoly over trade with Russian territory.^33^ The East India Company, chartered in 1600, was given similar privileges from the Cape of Good Hope to the Straits of Magellan.^34^ Over the next couple of decades, various additional business corporations were created by royal charter and granted monopolistic privileges.^35^ In the absence of any publicly available information regarding financial results and share prices, and without any sort of organised public market for shares, social networks involving mercantile elites provided sufficiently credible reassurance to induce investment in such firms and to underpin ‘secondary’ trading after companies distributed shares initially.^36^ Between the 1630s and the late 17th century, however, few new corporations were formed by royal charter and many existing companies atrophied or relinquished their monopoly privileges.^37^ Facilities for trading shares remained primitive, with market making in securities not having become a full-time vocation and with share dealing being confined mainly to well-connected investors linked to established mercantile networks.^38^

As the 17th century drew to a close, the UK experienced its initial substantial wave of company formations yielding a market for company shares.^39^ Company formation methods had evolved by this time, with Acts of Parliament largely supplanting royal charters and with various joint stock companies being established for the first time without reference to any form of explicit state endorsement or involvement.^40^ The latter approach was viable because of governmental reluctance to use prerogative writs of *quo warranto* and *scire facias* that were available to compel those operating enterprises without a charter or statutory authorisation to show why their unauthorised companies should not be dissolved.^41^

Of the nearly 150 companies in existence in England and Scotland as of 1695, approximately 85% had been launched since 1688.^42^ By this time, a fully fledged secondary market for company shares had developed,^43^ with Exchange Alley, a network of coffee houses in London, serving as the primary venue for share dealing.^44^ This occurred in the absence of a meaningful regulatory framework, either official or self-imposed by market practitioners.^45^

The initial flurry of company formations quickly subsided, and by 1698 less than one-third of the companies in existence in 1695 were in a position to carry on business.^46^ In 1697, Parliament enacted its first statute regulating equity markets, a ‘half-hearted’ initiative imposing restrictions on stockbrokers that expired in 1708.^47^ Otherwise, stock market-related activity lulled until the end of the 1710s, by which point only a tiny number of firms had any sort of active market for their shares.^48^ The situation then changed markedly, with a significant legislative legacy.

## 4. Bursting Bubbles (1720–1825)

Commentators have found fault with stock markets throughout their existence.^49^ A leading early critic was the famous novelist Daniel Defoe.^50^ In 1719, he described Exchange Alley as a ‘System of Knavery’ nourished by ‘false News’.^51^ Nevertheless, the equity market was starting to flourish that year, with 13 company ‘promotions’ (initial public offerings) occurring.^52^ The momentum accelerated in the first six months of 1720. Share prices of existing firms rose dramatically^53^ and 179 new companies were launched, many with an authorised share capital of £1 million or more.^54^ The number of individuals in the share market rose to 24,000, four times the mid-1690s figure.^55^

Opportunistic promoters responded to the stock market excitement by beginning to launch companies merely to profit from unloading shares on eager investors at the earliest opportunity.^56^ The ephemeral ventures involved were referred to derisively as ‘bubbles’.^57^ The enactment of legislation subsequently labelled ‘The Bubble Act’ set the scene for a share price crash in the second half of 1720 that brought the frenzy to an abrupt end with a panic that was ruinous for many investors, including Sir Isaac Newton.^58^

The Bubble Act prohibited, and prescribed penalties for, any undertaking that acted as a corporate body or raised capital by the issuance of transferable shares without specific authorisation from a statute or royal charter. LCB Gower said of the Bubble Act in the first edition of his classic company law text, ‘throughout the (18th) century and beyond the shadow of 1720 retarded the development of incorporated companies’.^59^ Numerous other commentators likewise maintain that the legislation substantially deterred the growth of corporate enterprise in the UK until its repeal in 1825.^60^ Larry Ribstein, an American corporate law scholar, has even argued the Bubble Act ‘throttled the British economy’.^61^

While the enactment of the Bubble Act does show that UK law makers could promulgate laws designed to hamper equity markets, it is doubtful whether the legislation had a substantial adverse impact on corporate enterprise. Britain experienced what is widely referred to as the Industrial Revolution in the second half of the 18th century and the opening decades of the 19th century.^62^ The manufacturing concerns at its heart rarely needed to contemplate adoption of the corporate form because they operated on a modest scale and could finance operations by drawing on personal funds, loans from family members, bank finance and cash injections from wealthy locals.^63^

The origins of the London Stock Exchange similarly indicate that the Bubble Act did not cripple corporate development. By the turn of the 19th century, dealing in securities was sufficiently well developed to prompt the 1801 launch of the LSE in its modern form, marked out by the establishment of a closed market where those trading became obliged to adhere to LSE rules and regulations governing how business would be conducted.^64^ An assortment of brokers and dealers additionally continued to trade shares outside ‘the House’.^65^

During the opening decades of the 19th century, the LSE was principally a secondary market for government debt securities.^66^ Nevertheless, 258 companies were being traded on the LSE at the time of the Bubble Act’s repeal.^67^ This was primarily because with businesses akin to public utilities, such as canals, water supply and docks, it was time consuming but feasible for proprietors to generate the public support necessary to secure parliamentary incorporation.^68^ Clauses in such private acts that defined for a company the roles and rights of shareholders and directors were ‘subject to the whims of its promoters’.^69^ Based, however, on legislation enacted in 1845 that drew on past practice in codifying clauses to be included in private incorporation acts, pre-1845 incorporation statutes likely included some meaningful protection for shareholders, such as providing for a right of first refusal when a company issued new shares (‘pre-emption rights’) and guaranteeing shareholders owning 10% or more of a company’s shares the right to call a shareholders’ meeting.^70^

In various additional sectors, such as mining and insurance, a deed of settlement mechanism was frequently used to achieve results mimicking full-scale incorporation through the creation of a joint stock company with a body of trustees that held the firm’s property for the benefit of investors.^71^ A company of this type technically contravened the Bubble Act because of the absence of a royal charter or statutory authorisation, but that legislation was only sporadically enforced.^72^ Ultimately, then, while the enactment of the Bubble Act demonstrates clearly that the UK has not always put out the welcome mat for publicly traded firms, the legislation did not fundamentally sidetrack the emergence of corporate enterprise in Britain.

## 5. Laissez-Faire Company Law (1825–1914)

The Bubble Act was an affirmative, if not particularly potent, barrier to the development of the publicly traded company in Britain. A different anti-stock market charge—counterproductive neglect of equity markets—can be laid against law makers from the mid-19th century through to the opening decades of the 20th century. During this era, regulation of publicly traded firms was rudimentary, with ‘caveat emptor the order of the day’.^73^

Companies legislation enacted between 1844 and 1862 simplified company formation considerably by providing a straightforward and reliable procedure to incorporate a company vested with essential corporate attributes such as full legal personality, transferable shares and, from 1855 onwards, limited liability for shareholders.^74^ By virtue of this legislation, ‘Britain had a laissez-faire company law regime’.^75^ Indices used by present-day empirical researchers to measure the quality of corporate law illustrate the point, with UK company law scores being very low by contemporary standards until well into the 20th century.^76^

The treatment of particular topics illustrates the rudimentary protection UK company law afforded investors. For instance, while regulation of prospectuses (documentation companies circulate in support of public offerings of shares) commenced in 1867 and was supplemented in 1890 by a liability regime, such regulation could be sidestepped readily until reforms in 1900 and 1907,^77^ and was lax compared with Germany’s regime.^78^ From 1856 through to 1900, UK companies legislation did not require that a company’s financial statements be audited.^79^ Only in 1908 did companies become obliged to file publicly their annual balance sheets.^80^ Also, until 1980 minority shareholders who believed they had suffered unjustified mistreatment at the hands of their company’s directors or dominant shareholders lacked a generally cast ‘unfair prejudice’ statutory mechanism under which they could seek relief.^81^ Even then, case law significantly eroded the scope to invoke the statutory mechanism in publicly traded companies.^82^

A case can be made that regulatory neglect had an adverse impact on Britain’s economy. While Britain was the pre-eminent industrial nation in the world during the middle decades of the 19th century, by World War I it was an also-ran compared to the United States and arguably Germany.^83^ Various commentators maintain that policy-maker neglect of the stock market was one cause.^84^

Britain, the argument goes, suffered in relative terms economically because in key industries such as automobiles, chemicals and electrical engineering, amateurish, family-dominated British firms came out second best to managerially adept American and German rivals. Ostensibly, those British firms that sought to keep pace with well-run international rivals struggled to raise sufficient capital on satisfactory terms because equity markets were ‘largely detached from manufacturing industry’.^85^ When companies did successfully access equity markets, this tended to coincide with periodic waves of ill-founded optimism that ended badly for many buyers of shares. Investors who lost out became prone to a ‘once burnt, twice shy’ mentality with domestic equities, making it increasingly difficult over time for worthwhile ventures to raise capital reliably on satisfactory terms. Misguided investor leaps in the dark were partly a product of substantial information asymmetries that a dearth of regulation of companies joining the stock market exacerbated.^86^

Seeking to link Britain’s economic decline to deficient equity markets arising from a laissez-faire regulatory regime is problematic in various ways. For instance, the theory that late 19th-century Britain ‘failed’ economically has been challenged forcefully.^87^ Certainly, losing out to the United States was something of an inevitability, given America’s much larger domestic market and the natural resources and human capital at its disposal.^88^ Also, it was understandable that British investors deployed capital liberally overseas rather than focusing exclusively on domestic concerns, given the beneficial opportunity to diversify risk and given the returns available elsewhere.^89^ Moreover, it is open to question whether British business was starved of capital: prior to the onset of World War I in 1914, industrial and commercial firms in the UK typically needed no more than a combination of retained earnings, bank lending and capital raised privately to operate successfully.^90^

Another reason to doubt that a laissez-faire company law regime contributed to a late 19th-century economic decline in Britain is that equity markets were well developed in various respects. There is broad agreement that the number of people owning shares grew substantially in the late 19th and early 20th centuries,^91^ backed by estimates of an increase from 250,000 investors to approximately 1 million between 1870 and the start of World War I.^92^ Moreover, by the mid-19th century, the London Stock Exchange was the biggest and most important stock market in the world,^93^ and it retained that status on the eve of World War I.^94^ Government and railway securities, both domestic and foreign, dominated, accounting in 1893 for nearly 71% of securities measured by paid-up capital, as compared to the 3.5% issued by commercial and industrial companies.^95^ Nevertheless, domestic industrial and commercial firms grew in prominence on the LSE as Britain ostensibly declined economically, accounting for 9.6% of the securities quoted by 1913.^96^

Stock exchanges operating in a number of ‘provincial’ (regional) centres such as Birmingham, Glasgow, Liverpool and Manchester buttressed equity markets further. As of 1869, there were more domestic companies listed on provincial stock exchanges than in London, a pattern which prevailed until the late 1890s even as the number of listings on the LSE increased.^97^ In sum, publicly traded firms seemingly had a considerably firmer foothold in corporate Britain than those who blame equity markets for the UK’s economic decline have assumed, with such firms often lacking a family or other dominant faction inclined to retain control of the business counterproductively instead of ceding managerial authority.^98^

But per the ‘law matters’ thesis, how did late 19th and early 20th-century UK equity markets grow when law makers were doing little to protect investors? The risks should have been well known, with various financial journalists and prominent novelists emphasising that money could be lost on the stock market, and fast.^99^ The UK’s elite investment banks perhaps could have helped to correct matters by acting as public offering gatekeepers. Instead, they generally eschewed domestic industrial companies seeking to raise capital to focus on lucrative work involving securities issuances by governments and railways. The market for domestic stock market flotations was therefore left to professional promoters, who often were little better than makeshift and at worst dishonest.^100^

What, then, did underpin demand for shares in UK equity markets from the late 19th century through to World War I? Shifts in market sentiment played a role, with investment ‘fads’ such as electricity companies, breweries, bicycles and rubber production periodically boosting demand for shares.^101^ More prosaically, stock exchanges can, independent of company law, impose constraints on those operating companies and protect investors by denying or revoking stock exchange listings with companies that fail to adhere to applicable listing rules.^102^ For a company seeking a quotation on the LSE during the late 19th and early 20th centuries, its prospectus had to be publicly advertised, it had to ensure that at least two-thirds of its shares would be distributed to the public and it had to have various provisions in its corporate constitution that conformed with Committee of the Stock Exchange guidance.^103^ These requirements may have gone ‘well beyond the flimsy protection given to shareholders by the early Limited Liability Acts’, but they were not ‘particularly severe by modern standards’.^104^ On the other hand, empirical research indicates the LSE vetoed numerous problematic applications for official quotations regardless of the rather limited grounds available for doing so.^105^

Provincial stock exchanges had their own listing rules which were similar to London’s, with substantial uniformity beginning to feature just before World War I.^106^ Different dynamics, however, may well have fortified demand for shares in regional equity markets.^107^ There was, reputedly, a sizeable, largely unacknowledged cohort of publicly traded firms where proprietors of industrial and commercial ventures arranged for the trading of shares on a provincial stock exchange to coincide with incorporation. Explicit investor protection was a secondary consideration because of a coterie of wealthy, often regionally based, initial shareholders well positioned to investigate relevant circumstances and look out for their own interests.

Private ordering likely underpinned stock market development in other ways too. In the late 19th century, it was commonplace for the corporate constitution of publicly traded firms to include provisions that afforded various rights to shareholders not mandated by companies legislation.^108^ Also, there was a strong norm among public companies in this era to make annual, stable dividend pay-outs, a practice from which investors could reasonably infer financial health because companies need cash on hand to pay dividends.^109^

## 6. War Time, and in Between (1914–45)

In 1914, government officials responded to the start of World War I by abruptly dropping the hands-off approach to equity markets that had prevailed previously and severely curtailed their operation. The same occurred when World War II got underway in 1939. During the intervening years, with respect to statutory regulation of publicly traded firms, the laissez-faire pre-World War I stance tended to prevail.

When World War I began, the Committee of the London Stock Exchange closed the Exchange.^110^ Those operating provincial stock exchanges did likewise. When the LSE and its provincial counterparts reopened on a limited scale after a few months, new issues of shares could only proceed if the Treasury and the relevant stock exchange committee gave the green light. With domestic companies, the formal test was whether a new issue was advisable in the national interest. Following an initial rush of applications, the Treasury, concerned that appeals to raise capital for private enterprise would erode support for the government’s war-driven fundraising, effectively put an end to public offerings of shares for the duration of the conflict. Throughout World War II, there were restrictions on capital raising akin to those in place during World War I.^111^

During the interwar era, two occurrences plausibly could have served as catalysts for the government to forsake its traditional hands-off approach and adopt policies to fortify equity markets. First, a sharp market downturn commencing in 1929 had adverse, sometimes scandal-ridden consequences. Second, a committee investigating banking, finance and credit chaired by Lord Hugh Macmillan identified in its 1931 report corporate finance deficiencies that would become known as the ‘Macmillan Gap’.^112^ Neither, however, sufficed to prompt a significant departure from the prevailing laissez-faire trend.

When share prices declined sharply in 1929 without rebounding meaningfully, a late-1920s new issue boom yielded disastrous returns for stock market investors.^113^ Scandal followed, with the business empire of well-known company promoter and financier Charles Hatry imploding despite illicit efforts to support share prices and the Royal Mail Steam Packet Company collapsing amidst false reporting of annual profits.^114^ Criticism of the stock market duly ensued. For instance, future Conservative Prime Minister Harold Macmillan drew on J Maynard Keynes’s 1936 classic *General Theory of Employment, Interest and Money* to write in 1938 ‘Mr Keynes tells us that day-to-day fluctuations of an ephemeral and non-significant character tend to have an altogether and even absurd influence on the market’.^115^

The LSE responded to the tumult by fortifying disclosure requirements in its listing rules and by warning the market that it was scrutinising applications for listing more closely than it had previously.^116^ There was no major legislative response in the 1930s, however.

The Companies Act 1929 implemented a 1926 report that was deferential to the laissez-faire status quo.^117^ With this legislation having been enacted so recently, the market turmoil that commenced the same year did not generate sufficient momentum to foster a company law overhaul. Parliament did ultimately enact the Prevention of Fraud (Investors) Act 1939, which prohibited unlicensed dealing in securities.^118^ However, this measure, which was not implemented until 1944, was designed as much to protect stockbrokers who were members of the LSE or a provincial stock exchange from unwelcome competition from legitimate ‘outside’ brokers catering to investors of modest means as it was to preclude fleecing by financial fraudsters.^119^

As for the Macmillan Gap, some context is required. Prior to World War I, as alluded to above, fledging industrial and commercial ventures often could obtain financial backing from affluent individuals, typically from the same region, with the trading of shares on a nearby provincial stock exchange being an entirely plausible outcome.^120^ High taxes on the wealthy imposed during World War I that were largely retained following the cessation of hostilities substantially impaired this finance model during the interwar years.^121^ For fledgling ventures wrong-footed by the tax-induced retreat of those who previously had the means to invest, securing long-term risk capital from conservative mainstream commercial banks on satisfactory terms was often impossible and high fees precluded relying on a reputable investment bank to secure equity financing by way of a public offering of shares.^122^ This was the financing ‘gap’ Lord Macmillan’s committee identified in its 1931 report.

The Macmillan Committee’s ideological predilections did much to preclude it from recommending state intervention to address the Macmillan Gap, with a core assumption being that financing the private sector was a matter for those whose business it was to deliver banking and financial services rather than the government.^123^ The Committee did suggest that there should be some special machinery in place to support relatively small domestic share issuances of between £5000 and £200,000.^124^ There was no official response, however, until 1945, when British banks, working under the leadership of the Bank of England and with governmental backing, formed and underwrote the Industrial and Commercial Financial Corporation to assist with the financing of small businesses.^125^

## 7. A Stock Market Dark Age (1945–79)

There is much discussion today about the stock market being under threat in the UK and a statistic cited to drive the point home is a substantial decline in the number of publicly traded companies.^126^ From this perspective, the stock market seemingly was in rude health in the decades immediately following World War II. The think tank New Financial evidenced the decline of the UK stock market in a 2019 article by pointing out that there were more than twice as many companies listed on the LSE in 1967 (3574) than in 2017 (1740).^127^ This in fact does not do full justice to the size of the post-World War II listed company cohort, since in March 1963 there were 4582 companies listed on the LSE.^128^ The number of individuals who owned shares had also increased, from an estimated 1.25 million in the late 1940s to 1.8 million in the mid-1960s.^129^ Healthy stock market returns likely served as a catalyst for this increase, with share prices of large companies rising 75% adjusted for inflation between 1951 and 1964.^130^

Despite some bright spots, due in large measure to government policy, the years between 1945 and 1979 were overall something of a stock market dark age. It is true the Companies Act 1948 gave shareholders a statutory right to vote by proxy and to dismiss directors mid-term, and, supplemented by the Companies Act 1967, expanded considerably the information that companies were obliged to divulge publicly.^131^ The 1967 legislation additionally tightened regulation of related party transactions that corporate insiders can rely on to extract private benefits of control.^132^ Still, while investors may have welcomed changes designed to ensure shareholders and creditors had access to information thought to be reasonably required and to make it easier for shareholders to exercise control over management,^133^ the policy rationale here was not to bolster the stock market per se. Instead, the 1948 and 1967 measures were both enacted by Labour governments, senior members of which ‘frequently denigrated the Stock Exchange as a glorified casino’.^134^

Nationalisation of industry was a significant post-World War II public policy blow to the stock market. The Labour government of 1945–51 took into state ownership coal mining (1946), civil aviation (1946), transport (eg railways and road haulage) (1947), electricity (1948) and gas (1948). Prior to the 1980s, the only reversals were steel and road haulage, and steel was renationalised in 1967.^135^ Nationalisation thus ‘removed whole categories of securities from those traded on the London Stock Exchange’, including railways, a stock market staple for over a century,^136^ and shrank the size of the stock market by one-third.^137^ In 1949, the *Financial Times* said the programme provided clear evidence of ‘The progressive deterioration in the investor’s status under the present Government’.^138^

With the post-World War II Conservative party leadership sharing many of the ‘dirigisme’ instincts of Labour,^139^ nationalisation was just one of a number of ‘compound disasters’ afflicting the mid-20th-century stock market.^140^ Another was that, from 1946 to 1958, Treasury consent was required for a company to raise more than £10,000 of equity capital in a calendar year.^141^ Moreover, dividend controls precluding companies from substantially increasing pay-outs to shareholders operated for most years between the mid-1960s and the end of the 1970s.^142^ On the tax front, for those with substantial incomes, a combination of very high marginal rates and the denial of deductions available for other forms of income meant that, for individuals who owned shares, the after-tax return from dividends was negligible.^143^ Also, in 1965, the government imposed capital gains tax on profits derived from selling shares at a level (30%) that was high compared with rates in other countries.^144^

There was widespread awareness that post-World War II government policies were antithetical to stock market investment. In 1962, the chair of the LSE suggested that the government should focus on removing ‘restrictions and barriers’ rather than ‘thinking up new schemes that will restrict and hamper business’.^145^*The Telegraph* newspaper cited high taxation when remarking in 1977 on ‘the last stand of the small investor’, who was ‘threatened with extinction’.^146^ The LSE did likewise in a 1977 report to a government committee, where it noted that the private investor had been ‘a consistent net seller of securities’.^147^ The proportion of shares in UK publicly traded companies individuals owned directly indeed fell from 65.8% in 1957 to 28.2% in 1981.^148^

The implications of post-World War II governmental policies for the stock market could have been truly dire if domestic institutional investors had not come to the rescue by taking the place of retail investors as buyers of shares.^149^*The Economist* said of the transition in 1975 ‘Usually in this London of the last days of the rich, private individuals are net sellers of stock which the institutions buy’.^150^ UK-based pension fund and insurance companies in particular had ample funds on hand to deploy and could, in contrast to individuals, hold equities in a manner advantageous from a tax perspective.^151^ These investors were sufficiently impressed with domestic stock market returns in the 1950s and 1960s for an institutional ‘cult of the equity’ to develop.^152^ The proportion of UK public company shares pension funds and insurance companies owned duly increased from a combined 12.2% in 1957 to 47.2% in 1981.^153^

Even with domestic institutional shareholders riding to the rescue to some degree, the stock market was in a pretty dismal state by the end of the 1970s. For a country, if the ratio of the aggregate market capitalisation of publicly traded shares to gross domestic product (GDP) is 1:1 or greater, this implies the stock market is of substantial importance in relation to the economy.^154^ Historical data for the UK can vary widely for particular time periods,^155^ but there is a consensus that as the 1970s drew to a close the aggregate market capitalisation/GDP ratio was well below 1:1.^156^ In contrast, the ratio was substantially greater than 1:1 as the 20th century got underway,^157^ and has usually exceeded 1:1 since the mid-1990s.^158^

## 8. A Stock Exchange Golden Era (Mostly) (1980–2000)

In contrast with what was in various ways a stock market dark age between 1945 and 1979, 1980 to 2000 was akin to a stock market golden era. The decline in the number of companies traded on the London Stock Exchange continued as the 20th century drew to a close. Nevertheless, the fall was only marginal, from 3141 in 1980 to 2929 in 2000, as compared to a marked decline from nearly 4600 in 1963 to 3190 in 1979.^159^

On the upside, the UK stock market delivered well for investors compared to global peers.^160^ Shares also easily outperformed government bonds and, as the 20th century drew to a close, ‘inflation-busting returns were the norm: between 7 and 10 per cent a year’.^161^ The buoyant stock market helped to underpin a substantial increase in the UK’s aggregate market capitalisation/GDP ratio.^162^ The pattern substantiates business historian Leslie Hannah’s claim that, during the final quarter of the 20th century, a stock market reversal was itself reversed, meaning Britain was once again ‘one of the most substantially stock exchange-driven economies in the world’.^163^

Various pro-stock market policy moves accompanied and fortified the stock market revival. Margaret Thatcher’s Conservative party cancelled dividend controls almost immediately after coming to power in 1979.^164^ The Thatcher administration subsequently substantially dismantled the harsh tax bias that discouraged individual share ownership.^165^ It also created the Personal Equity Plan to offer individuals fresh tax concessions to foster long-term shareholdings.^166^ For large financial institutions, the transaction costs associated with equity investment declined considerably when, as part of a thoroughgoing mid-1980s government-backed reorganisation of the financial services industry known as ‘Big Bang’, fixed share-dealing commissions were abolished.^167^

The LSE, which obtained in 1973 a *de facto* UK monopoly over dealing in publicly traded shares when all British stock exchanges merged into a single entity,^168^ contributed to the pro-stock market policy momentum when it established the Unlisted Securities Market (USM) in 1980.^169^ The move was prompted by a government committee investigating the functioning of financial institutions, which recommended in a 1979 interim report that the LSE set up a market for the trading of shares of companies not inclined to seek out a full LSE quotation.^170^ After its launch, the USM would create something of a stock market ‘buzz’.^171^ Ultimately, it was deemed a 1980s success^172^ that was ‘a symbol of the Thatcherite enterprise culture’.^173^

A privatisation campaign the Conservative government launched was the most potent policy move during the Thatcher era linked to the stock market’s revival. Privatisation kicked into high gear in 1984 with the sale of 51% of the shares of British Telecom to public investors.^174^ Ultimately, privatisation would be for corporate Britain the policy that would ‘always be associated with’ Thatcher’s tenure in office,^175^ would reduce the share of employment for which British nationalised industries accounted from 9% to under 2%^176^ and would be imitated worldwide.^177^

The Thatcher government’s privatisation campaign had various goals. One was to generate revenue to help to balance the government’s books.^178^ Another was to shift management from the state’s hands to private ownership to seek to unlock efficiency gains and improve service quality.^179^ An additional objective had direct implications for the stock market, namely broadening share ownership.

The Conservatives assumed that voters who had a direct financial stake in British business were likely to support the market-friendly agenda the Thatcher administration was seeking to implement.^180^ Privatisation accordingly was ‘used vigorously as a device for widening share ownership’,^181^ with substantial advertising and generous share purchase terms being deployed to foster demand from retail investors.^182^ The number of individuals in the UK owning shares duly grew from 3 million when Thatcher became Prime Minister in 1979 to 11 million in 1990 when she left office.^183^ ‘[T]he government’s hopes of creating a larger body of little capitalists’^184^ thus seemed to be realistic.

While the privatisation campaign did provide a meaningful boost to the stock market by returning most nationalised industries to private ownership, ultimately ‘Mrs Thatcher’s hope of a share-owning democracy never really took off’.^185^ In general terms, for retail investors, collective investment vehicles such as unit trusts were a more sensible and convenient equity ownership option than holding shares directly in particular companies.^186^ Moreover, many individuals who bought shares in generously priced privatisation initial public offerings turned a quick profit by selling out immediately.^187^ For those who did not exit promptly, a sharp drop in share prices in 1987 shook the confidence of many.^188^ A by-product was that only a small minority of those who purchased shares during 1980s privatisations went on to buy shares directly in any additional companies.^189^ This all meant that, despite privatisation’s emphasis on broadening share ownership, the proportion of shares in UK publicly traded companies that individuals owned continued to fall, dropping to 20.6% in 1989.^190^ There was a further drop to 15.3% in 1999,^191^ despite additional privatisations in the 1990s.^192^

The pattern was similarly uneven with the provision of a market for the trading of shares of companies for which a full quotation on the LSE was not on the cards. The USM may have been a good fit with the 1980s’ entrepreneurial zeitgeist, but by the early 1990s it was widely regarded as a ‘dying market’.^193^ There were, for instance, merely seven new issues on the USM in 1991, compared with 103 in 1988.^194^ Reasons for the USM’s sharp decline included depressed market sentiment associated with an early-1990s recession and a European Union capital market reform-inspired narrowing of the regulatory requirements involved with joining the USM and with obtaining a full LSE quotation, with the latter being preferable due to greater prestige and visibility.^195^ The LSE announced in late 1992 it was minded to phase out the USM by 1995.^196^ It confirmed its intention in 1993.^197^

The London Stock Exchange’s original plan was that USM companies would have two options after the USM closed, namely obtaining a full quotation or losing their LSE trading status entirely.^198^ The LSE rethought its position when the Confederation of Business Industry and financial services professionals closely linked with the small companies sector lobbied in favour of a USM replacement, a stance John Major’s Conservative government supported.^199^ The LSE picked up on the cue, publishing in February 1995 detailed rules for the Alternative Investment Market (AIM),^200^ which opened on schedule in June of that year.^201^ Flouting predictions of AIM’s early demise,^202^ an optimistic target of 140 companies joining in its first year was exceeded.^203^ AIM thus surprised pessimists and even many of its supporters.^204^ AIM would additionally prove to have more staying power than the USM, with a 2007 London School of Economics study remarking ‘the success of AIM has prompted imitation, envy and criticism from around the world’.^205^

## 9. Challenges Mount (2000–20)

While the 1980s and 1990s were in various respects a golden age for the stock market in the UK, criticism of the stock exchange grew, and did so in a way that would compound challenges mounting during the opening decades of the 21st century. Corporate Britain experienced ‘mega-merger mayhem’ in the mid- and late 1980s.^206^ A growing band of academics voiced concerns that publicly traded firms fearful of being lost in the acquisition shuffle were counterproductively forsaking long-term success to keep onside investors interested only in short-term earnings data and the next dividend pay-out.^207^ Various corporate executives and public officials,^208^ including Nigel Lawson,^209^ the Chancellor of the Exchequer from 1983 to 1989, offered varying degrees of support for this increasingly popular negative stock market verdict.^210^

Stock market misgivings would ultimately become influential in the public policy realm, and in ways that arguably had an adverse impact on the stock market as it was facing post-2000 headwinds. In 1994, the financial secretary to the Treasury expressed concern about the high level of dividends companies were paying out to shareholders, a view the Labour opposition shared.^211^ Labour, having come to power in 1997, promptly cancelled a right that pension funds had, as tax-exempt investors, to reclaim dividend tax credits, thereby reducing the value of their dividend income by 20%.^212^ Gordon Brown, Labour’s Chancellor of the Exchequer, justified the move on the basis that firms would be able to deploy increased retained earnings beneficially to finance future growth because pension funds would no longer be demanding tax-subsidised dividends.^213^ For pension funds, the change in policy was one cause of a rapid exit from UK shares.^214^ Between 1996 and 2006, they reduced the portion of their investment portfolios allocated to UK equities from 58% to 33%.^215^

The pension fund sell-off was just one of a series of setbacks the UK stock market suffered as the 21st century got underway. For instance, regulatory changes designed to fortify insurer financial buffers prompted domestic insurance companies to join pension funds in exiting from UK equities.^216^ The combined stake domestic pension funds and insurers held in UK quoted companies correspondingly fell precipitously from 50% in 1994 to 11% in 2012.^217^ The exit by these erstwhile stock market stalwarts resulted in share registers of publicly traded companies being ‘split between an increasingly complex array of foreign and domestic investors’.^218^ There additionally were doubts about where demand for UK equities would come from in the future.^219^

Share prices were also in the doldrums. Between 1997 and 2004, the FTSE 100 stock market index increased a mere 2.1% unadjusted for inflation, compared with 42% for France’s CAC 40 index and 49.8% for America’s blue-chip Dow Jones Industrial index.^220^ The trend persisted through to the early 2020s, with key UK share price indices consistently lagging peers in the rest of the developed world.^221^

As for the number of companies traded on the London Stock Exchange, this was higher in the mid-2000s than in 1999,^222^ primarily due to ‘exceptional growth’ in the number of companies traded on AIM.^223^ From the late 2000s onwards, however, the number of AIM listings declined amidst pessimistic speculation that the junior market could end up in ‘the last chance saloon’.^224^ The total number of companies traded on the LSE in turn fell steadily.^225^ Given this, and given flat share prices, an aggregate market capitalisation/GDP ratio that peaked in 1999 at 1.75:1 went unsurpassed subsequently.^226^

The LSE became sufficiently concerned about stock market trends to commission in 2004 an internal report on the erosion of the UK’s equity culture.^227^ The government’s response was considerably more ambivalent. This was the case despite the ostensibly market-friendly Conservatives returning to power in 2010 in a coalition with the Liberal Democrats and despite growing awareness the UK equity market was becoming ‘a relative backwater’.^228^

On one hand, public officials were favourably disposed toward a move to attract firms with strong overseas connections to London. In 2000, a statutorily backed regulator, the Financial Services Authority (FSA), the predecessor of the FCA, took over the role of the UK Listing Authority from the LSE, meaning the FSA became responsible for promulgating and enforcing the listing rules that governed companies quoted on the LSE’s Main Market.^229^ In the mid-2000s, the LSE successfully lobbied numerous overseas-based enterprises to list in London.^230^ The FSA provided a helping hand by using its discretion to relax initial listing requirements for various high-profile quoted sector entrants that were not fully compliant.^231^ This, however, proved to be a faulty way to bolster UK equity markets, with governance scandals in the early 2010s afflicting the overseas listings cohort in a way that cast doubt on the credibility of listed company regulation.^232^

In contrast with the FSA’s efforts to encourage listings in London, reinvigorating the stock market was not an obvious priority with a major review of equity markets the Conservative-led coalition government commissioned in 2011. Vince Cable, the business secretary, appointed economist John Kay to conduct the review.^233^ Cable had told the 2010 Liberal Democrat conference that markets were ‘often irrational or rigged’, and intimated that ‘good companies’ traded on the stock market were frequently ‘destroyed by short term investors looking for a speculative killing’.^234^ Cable was thus unlikely to give the assignment to review UK equity markets to an unvarnished stock market booster. Kay, indeed, had expressed stock exchange-related misgivings throughout his career. For instance, in the 1980s, he had suggested that privatisations were unlikely to improve the managerial culture of the companies affected in the manner the government assumed.^235^ He went on to argue in the 1990s that the diffuse share ownership UK stock market companies often featured discouraged the fostering at firm level of beneficial, sustained trust-based relationships that typically underpin enduring corporate success.^236^ Moreover, Kay acknowledged when he launched his review of equity markets that he had become increasingly sceptical over time of the efficacy of stock market pricing.^237^

Kay noted in his 2012 final report that the number of companies traded on the LSE had been declining steadily.^238^ Nevertheless, consistent with the views he and Cable had expressed previously, he acknowledged frankly that the review was not an exercise intended to bolster the stock market. Instead, ‘We do not believe there are any arguments of policy for promoting the use of public equity markets as an objective in itself’.^239^

Kay highlighted various stock market shortcomings in his review. For instance, he drew attention to instances where short-term thinking by those running British publicly traded companies had led to bad long-term decisions that shareholder engagement counterproductively tended to foster rather than discourage.^240^ He also emphasised that equity markets are not, as is often assumed, an important source of capital for new investment for publicly traded firms.^241^ Kay said in addition that a decade of disappointing UK stock market returns had disenchanted investors.^242^ He noted as well that companies that were publicly traded often resented burdensome corporate governance obligations.^243^

The Kay Review did acknowledge that there should not be ‘unnecessary disincentives to using equity markets’.^244^ Still, given Kay’s stock market scepticism, it would have been incongruous if he had made recommendations intended specifically to boost the stock market. Kay focused instead on proposals designed ‘to support sustainable long-term value creation by British companies’, such as restoring ‘relationships of trust and confidence in the investment chain’ between end investors and companies, and ‘Improving the quality of engagement by investors with companies’.^245^ The UK government welcomed the Kay Review’s final report and undertook to make feasible changes to regulation to implement directions for regulatory policy Kay had offered.^246^

Less than a decade after the UK government endorsed a report that was explicitly ambivalent regarding the promotion of equity markets, Rishi Sunak, as Chancellor of the Exchequer, told Parliament in November 2020 of the government’s desire ‘To boost the number of new companies that want to list here in the UK’ and to make ‘sure that our listings regime is as competitive as it can be to make sure that we attract companies to list here in London’.^247^ Following on from a government-commissioned review of UK equity markets, the FCA amended its Listing Rules in 2021 to relax various requirements companies seeking to list had faced, most prominently in relation to the allocation of voting rights, the level of shares in public hands (the ‘free float’) and the deployment of special acquisition companies (SPACs) to bring firms to the stock market.^248^ The FCA returned to the deregulation theme in 2023 as it proposed eliminating a listing rule distinction between ‘standard’ and ‘premium’ tier companies with a view to simplifying or abolishing various requirements imposed solely on ‘premium’ tier companies.^249^

Why the rethink? With the number of publicly traded companies continuing to decline as initial public offering activity dwindled, pleas by fund managers and quoted companies for pro-stock market change increasingly resonated.^250^ Brexit also played a significant role. Immediately following the 2016 referendum Brexit’s impact on London’s financial district was not a governmental priority, in part because ‘the City’ had backed ‘Remain’.^251^ The Conservative government belatedly realised, however, that Brexit had had an adverse impact on the City that put London’s status as a global financial centre in jeopardy,^252^ meaning ‘this national cash register (could) no longer be taken for granted’.^253^ Given this, and given that Brexiteers had maintained that a break with the European Union would create opportunities for beneficial regulatory change,^254^ a Brexit-related case for stock market-friendly reform took shape. The call for evidence that kicked off the reform process that culminated in the FCA’s 2021 revision of its Listing Rules indeed specifically referenced the Brexit-related opportunity to recalibrate equity market regulation to fit the needs of ‘companies, investors and markets’ better.^255^

It is beyond the scope of this article to assess the listing rule changes recently implemented and proposed. Two points, however, merit elaboration. First, the current direction of travel is different than the ‘law matters’ thesis concerning stock market development prescribes. The emphasis with current reforms is on relaxing existing requirements rather than bolstering investor protection,^256^ which is the ‘law matters’ prescription for robust equity markets. Correspondingly, if the current reforms do foster stock market development, this will not serve as a belated UK-related validation of the law matters thesis.

Second, even if the reforms do provide a boost to the UK’s equity markets, it is doubtful whether they will foster decisive change. Having made this point about the reforms the FCA proposed in 2023, *The Economist* explained ‘The reason is that the rot in Britain’s stockmarket goes far deeper than its rule book’, citing investor deficiencies in particular.^257^ The FCA has indeed acknowledged the challenge itself, saying, when announcing its 2023 Listing Rule proposals, that ‘changing the listing rules can only be one part of making the UK’s capital markets work better and will involve collective action to embrace greater risk’.^258^

## 10. Conclusion

An influential ‘law and finance’ thesis is that strong legal protection for investors is essential for a vibrant stock market to develop. Britain poses a challenge to this reasoning because it had thriving equity markets well before mid-20th-century statutory changes considerably bolstered regulation of publicly traded companies. As this article has indicated, the point can be put somewhat more strongly: since the stock market began to feature in economic life in the UK, public officials have periodically taken steps antithetical to stock exchange development. Otherwise, a hands-off stance tended to be the default position.

The tables have turned dramatically recently. Evidence that the UK stock market is in decline has mounted and public officials, somewhat belatedly realising that what has been a mainstay of the British economy is in jeopardy, have begun introducing reforms to try to reverse the trend. It remains to be seen whether this government-initiated effort to revive the stock market will succeed. As this article has shown, however, law and stock market development have been uneasy bedfellows in Britain, which implies that factors other than regulation will primarily dictate the future fortunes of the UK’s equity markets.

## Footnotes

1 HM Treasury, ‘UK Listings Review: Government Response, 21 April 2021’ <www.gov.uk/government/publications/uk-listings-review/uk-listings-review-government-response>. All URLs were last accessed on 1 July 2023 unless otherwise stated.

2 Ajit Singh, ‘Should Africa Promote Stock Market Capitalism?’ (1999) 11 Journal of International Development 343, 344.

3 Kieran Heinemann, *Playing the Market: Retail Investment and Speculation in Twentieth-century Britain* (OUP 2021) 228. See also Allen Sykes, ‘Proposals for Internationally Competitive Corporate Governance in Britain and America’ (1994) 2 *Corporate Governance: An International Review* 187, 188; Bernard S Black, ‘The Core Institutions that Support Strong Securities Markets’ (2000) 55 Bus Law 1565, 1565.

4 Ranald C Michie, *The London Stock Exchange: A History* (OUP 1999) 15; Jonathan P Charkham, *Keeping Better Company: Corporate Governance Ten Years On* (OUP 2004) 305–6.

5 ‘From Big Bang to a Whimper’ *The Economist* (2 October 2021) 9.

6 ‘UK Listings Review’ <www.gov.uk/government/publications/uk-listings-review>.

7 ibid.

8 On the proposals and FCA implementation, see Brian R Cheffins and Bobby V Reddy, ‘Will Listing Rule Reform Deliver Strong Public Markets for the UK?’ (2023) 86 MLR 176, 190–2.

9 FCA, *Primary Markets Effectiveness Review: Feedback to DP22/2 and Proposed Equity Listing Rule Reforms* (FCA 2023) 3, 6.

10 James R Brown, Gustav Martinsson and Bruce C Petersen, ‘Law, Stock Markets, and Innovation’ (2013) 68 Journal of Finance 1507, 1517. For examples, see Black (n 3); Rafael La Porta and others, ‘Law and Finance’ (1998) 106 *Journal of Political Economy* 1113.

11 See eg Peter Harrison, ‘The City Must Back Sunak’s Vision for the Stock Market’ *Sunday Times* (London, 11 April 2021); Philip Augar, ‘“Big Bang 2” Reforms Expose the City’s Weaknesses’ *Financial Times* (25 November 2022).

12 See eg Black (n 3) 1565; Brown, Martinsson and Petersen (n 10) 1544.

13 See eg Mats Isaksson and Serdar Çelik, ‘Who Cares? Corporate Governance in Today’s Equity Markets’ (2013) OECD Corporate Governance Working Papers No 8, 10 (discussing the OECD’s stance).

14 On the fact the reversal is largely forgotten, see Leslie Hannah, ‘London as the Global Market for Corporate Securities before 1914’ in Laure Quennouëlle-Corre and Youssef Cassis (eds), *Financial Centres and International Capital Flows in the Nineteenth and Twentieth Centuries* (OUP 2011) 149.

15 For classic histories, see Michie, *The London Stock Exchange* (n 4); E Victor Morgan and WA Thomas, *The Stock Exchange: Its History and Functions* (2nd edn, Elek Books 1969). For examples of more recent work, see Heinemann, *Playing the Market* (n 3); Amy Edwards, *Are We Rich Yet? The Rise of Mass Investment Culture in Contemporary Britain* (University of California Press 2022).

16 For recent examples, see Timothy W Guinnane, Ron Harris and Naomi R Lamoreaux, ‘Contractual Freedom and Corporate Governance in Britain in the Late Nineteenth and Early Twentieth Centuries’ (2017) 91 Business History Review 227; Christopher Coyle, Aldo Musacchio and John D Turner, ‘Law and Finance in Britain *c* 1900’ (2019) 26 Financial History Review 267.

17 An Act for better securing certain Powers and Privileges, intended to be granted by His Majesty by Two Charters, for Assurance of Ships and Merchandise at Sea, and for lending Money upon Bottomry; and for restraining several extravagant and unwarrantable Practices (6 Geo 1 c 18), ss 18–21.

18 Black (n 3) 1565.

19 On the literature and its popularity, see John Armour and others, ‘How Do Legal Rules Evolve? Evidence from a Cross-Country Comparison of Shareholder, Creditor and Worker Protection’ (2009) 58 Am J Comp L 579, 582–5.

20 See Mark Roe, ‘Corporate Law’s Limits’ (2002) 31 JLS 233, 236–7; Luca Enriques, ‘Do Corporate Law Judges Matter? Some Evidence from Milan’ (2002) 3 EBOR 756, 766–7.

21 The terminology is borrowed from Mark J Roe, ‘Political Preconditions to Separating Ownership from Corporate Control’ (2000) 53 Stan L Rev 539, 585.

22 Black (n 3) 1567–8, 1570–3, 1576, 1578, 1583–7; La Porta and others (n 10) 1126–34; Rafael La Porta, Florencio Lopez-de-Silanes and Andrei Shleifer, ‘What Works in Securities Laws?’ (2006) 61 Journal of Finance 1; Simeon Djankov and others, ‘The Law and Economics of Self-Dealing’ (2008) 88 Journal of Financial Economics 430.

23 Diane K Denis, and John J McConnell, ‘International Corporate Governance’ (2003) 38 Journal of Financial and Quantitative Analysis 1, 30.

24 Clifford Holderness, ‘Law and Ownership Re-examined’ (2016) 5 Critical Finance Review 41, 42.

25 See eg Holger Spamann, ‘The “Antidirector Rights Index” Revisited’ (2010) 23 Review of Financial Studies 467.

26 Holderness (n 24) 45.

27 Djankov and others (n 22) 461–2. cf Holderness (n 24) 59 (acknowledging the concession by Djankov and co-authors, but saying the self-dealing index did not perform as well in his regressions).

28 Coyle, Musacchio and Turner (n 16) 268.

29 ibid; Brian R Cheffins, ‘Does Law Matter?: The Separation of Ownership and Control in the United Kingdom’ (2001) 30 JLS 459; Graeme G Acheson and others, ‘Corporate Ownership and Control in Victorian Britain’ (2015) 68 Economic History Review 911, 912–13, 915.

30 Coyle, Musacchio and Turner (n 16) 268.

31 ibid 286.

32 Brian R Cheffins, *Corporate Ownership and Control: British Business Transformed* (OUP 2008) 106–15, 119–21.

33 William R Scott, *The Constitution and Finance of English, Scottish and Irish Joint-Stock Companies to 1720*, vol 1 (CUP 1912) 17–19; Ron Harris, *Industrializing English Law: Entrepreneurship and Business Organization, 1720–1844* (OUP 2000) 33, 43.

34 Harris (n 33) 43–4.

35 ibid 45.

36 Edmond Smith, ‘The Social Networks of Investment in Early Modern England’ (2021) 64 Historical Journal 912.

37 Harris (n 33) 46–8.

38 KG Davies, ‘Joint Stock Investment in the Later Seventeenth Century’ (1952) 4 Economic History Review 283, 294–5.

39 Cheffins, *Corporate Ownership and Control* (n 32) 134.

40 Harris (n 33) 53.

41 ibid; Cheffins, *Corporate Ownership and Control* (n 32) 135.

42 Scott (n 33) 327.

43 Ann Carlos, Larry Neal and Kirsten Wandschneider, ‘Networks and Market Makers in the First Emerging Market: Bank of England Shares, London 1720’ (2007) unpublished working paper, 2.

44 Stuart Banner, *Anglo-American Securities Regulation and Political Roots, 1690–1860* (CUP 1998) 24.

45 Richard Dale, *The First Crash: Lessons from the South Sea Bubble* (Princeton University Press 2004) 33.

46 Scott (n 33) 356.

47 Banner (n 44) 39–40.

48 Harris (n 33) 57–8.

49 Catherine R Macaulay, ‘Financializing Capitalism: 400 Years of Equity Market Development’ (2019) 23 Competition & Change 502, 503.

50 Banner (n 44) 32.

51 Daniel Defoe, *The Anatomy of Exchange-Alley: Or, a System of Stock-Jobbing* (E Smith near Exchange-Alley 1719) 3.

52 William R Scott, *The Constitution and Finance of English, Scottish and Irish Joint-Stock Companies to 1720*, vol III (CUP 1912) 445.

53 Ann Carlos, Erin Fletcher and Larry Neal, ‘Share Portfolios in the Early Years of Financial Capitalism: London, 1690–1730’ (2015) 68 Economic History Review 574, 582–3.

54 Scott (vol III) (n 52) 446–57.

55 Carlos, Fletcher and Neal (n 53) 589–90.

56 Cheffins, *Corporate Ownership and Control* (n 32) 138.

57 Charles Mackay, *Memoirs of Extraordinary Popular Delusions and the Madness of Crowds* (Robson, Levey and Franklin 1852) chs 2, 15.

58 Dale (n 45) 135–6; John Carswell, *The South Sea Bubble* (rev edn, Alan Sutton 1993) 129–30, 141–2, 161–5.

59 LCB Gower, *The Principles of Modern Company Law* (Stevens & Sons 1954) 31.

60 For examples, see Harris (n 33) 61, fn 4. See also Macaulay (n 49) 506; George W Edwards, *The Evolution of Financial Capitalism* (Longmans, Green & Co 1938) 14; Hamish McRae and Frances Cairncross, *Capital City: London as a Financial Centre* (Methuen 1991) 10–11.

61 Larry E Ribstein, *The Rise of the Uncorporation* (OUP 2010) 91.

62 Joel Mokyr, ‘Editor’s Introduction: The New Economic History and the Industrial Revolution’ in Joel Mokyr (ed), *The British Industrial Revolution: An Economic Perspective* (2nd edn, Routledge 1999) 3.

63 Brian R Cheffins, ‘Law, the Market and Corporate Enterprise’ in John Armour and Jennifer Payne (eds), *Rationality in Company Law: Essays in Honour of DD Prentice* (Hart Publishing 2009) 313–15; Ryan Bubb, ‘Choosing the Partnership: English Business Organization Law During the Industrial Revolution’ (2015) 38 Seattle U L Rev 337, 353–6.

64 Michie, *The London Stock Exchange* (n 4) 34–7.

65 James Taylor, ‘Inside and Outside the London Stock Exchange: Stockbrokers and Speculation in Late Victorian Britain’ (2021) 22 Enterprise & Society 842, 846–7.

66 Harris (n 33) 119.

67 ibid 219, Table 8.1.

68 ibid 97–9; 219–22; Mark Freeman, Robin Pearson and James Taylor, *Shareholder Democracies: Corporate Governance in Britain and Ireland Before 1850* (University of Chicago Press 2011) 24, 42, 47–9.

69 Frank A Sharman, ‘The History of the Lands Clauses Consolidation Act 1845—I’ [1986] Stat LR 13, 19.

70 Companies Clauses Consolidation Act 1845, c 16, ss 58, 70 <www.legislation.gov.uk/ukpga/Vict/8-9/16/contents>; James Foreman-Peck and Leslie Hannah, ‘UK Corporate Law and Corporate Governance before 1914: a Re-interpretation’ (2015) EHES Working Papers in Economic History No 72, 6. Foreman-Peck and Hannah argue that the 1845 legislation merited a high score of 5 out of 6 on the ADRI (p 9). Others award the CCCA a much more modest 3: Coyle, Musacchio and Turner (n 16) 272–3; Graeme G Acheson, Gareth Campbell and John D Turner, ‘Private Contracting, Law and Finance’ (2019) 32 Review of Financial Studies 4156, 4159. The 1845 Act also did not regulate related party transactions, nor did it mandate disclosure of information when new shares were issued, laws that the ADRI’s creators subsequently acknowledged were of greater significance (n 27 and related discussion).

71 Bubb (n 63) 349–50; Freeman, Pearson and Taylor (n 68) 53–4. There is disagreement on the extent to which the deed of settlement model was an adequate substitute for statutorily authorised incorporation. Compare Bubb (n 63) 345–9 (arguing the model was an effective substitute) with Harris (n 33) 141–59; Andreas Televantos, *Capitalism before Corporations: The Morality of Business Associations and the Roots of Commercial Equity and Law* (OUP 2020) 43–6, 51–2.

72 Freeman, Pearson and Taylor (n 68) 28–9, 53; John Morley, ‘The Common Law Corporation: The Power of the Trust in Anglo-American Business History’ (2016) 116 Colum L Rev 2145, 2158–9.

73 David Kynaston, *The City of London, Vol II: Golden Years, 1890–1914* (Chatto & Windus 1995) 459.

74 Joint Stock Companies Act 1844 (7 & 8 Vict c 110); Limited Liability Act 1855 (18 & 19 Vict c 133); Joint Stock Companies Act 1856 (19 & 20 Vict c 47); Companies Act 1862 (25 & 26 Vict c 89), summarised by Cheffins, *Corporate Ownership and Control* (n 32) 164–5.

75 Graeme G Acheson and others, ‘Corporate Ownership and Control in Victorian Britain’ (2015) 68 Economic History Review 911, 912.

76 ibid 915.

77 Companies Act, 1867 (30 & 31 Vict c 131), s 38; Directors Liability Act, 1890 (53 & 54 Vict c 64); Bernard J Kilbride, ‘The British Heritage of Securities Legislation in the United States’ (1963) 17 SMU L Rev 258, 266; Louise Gullifer and Jennifer Payne, *Corporate Finance Principles and Policy* (3rd edn Hart Publishing 2020) 520.

78 Carsten Burhop, David Chambers and Brian R Cheffins, ‘Regulating IPOs: Evidence from Going Public in London and Berlin, 1900–1913’ (2012) University of Cambridge Faculty of Law Legal Studies Research Papers No 2/2012, 11–12.

79 Cheffins, *Corporate Ownership and Control* (n 32) 172, 194.

80 ibid.

81 Companies Act 1980 (c 22), s 75, now Companies Act 2006 (c 46), s 994.

82
*Re Blue Arrow plc* [1987] ChD 585; *Re Tottenham Hotspur plc* [1994] 1 BCLC 655; *Re Astec (BSR) plc* [1998] 2 BCLC 556, 589.

83 Alfred D Chandler, *Scale and Scope: The Dynamics of Industrial Capitalism* (Belknap Press 1990) 3–4.

84 William Kennedy has been the leading proponent of this theory. See William P Kennedy, ‘Book Review’ (1983) 43 Journal of Economic History 750; William P Kennedy, ‘Notes on Economic Efficiency in Historical Perspective: The Case of Britain, 1870–1914’ (1984) 9 Research in Economic History 109; William P Kennedy, *Industrial Structure, Capital Markets and the Origins of British Economic Decline* (CUP 1987). See also Chandler (n 83) 240–2, 291–4, 389–92; Sidney Pollard, ‘Capital Exports, 1870–1914: Harmful or Beneficial?’ (1985) 38 Economic History Review 489, 512–14; J Bradford De Long, ‘Did Morgan’s Men Add Value? An Economist’s Perspective on Financial Capitalism’ in Peter Temin (ed), *Inside the Business Enterprise: Historical Perspectives on the Use of Information* (University of Chicago Press 1991) 229–30.

85 Geoffrey Owen, *From Empire to Europe: The Decline and Revival of British Industry Since the Second World War* (Harper Collins 1999) 393.

86 This pessimistic characterisation of late 19th and early 20th-century UK equity markets is developed most fully by John Armstrong, ‘The Rise and Fall of the Company Promoter and the Financing of British Industry’ in JJ van Helten and Y Cassis (eds), *Capitalism in a Mature Economy: Financial Institutions, Capital Exports and British Industry, 1870–1939* (Edward Elgar 1990). Armstrong only alludes very briefly, however, to the possibility that the pattern he describes may have contributed to Britain’s economic decline—see 133–4.

87 The best-known critique is Donald N McCloskey, ‘Did Victorian Britain Fail?’ (1970) 23 Economic History Review 446.

88 Nicholas Crafts, *Forging Ahead, Falling Behind and Fighting Back: British Economic Growth from the Industrial Revolution to the Financial Crisis* (OUP 2021) 50–1, 58.

89 ibid 48; Benjamin R Chabot and Christopher J Kurz, ‘That’s Where the Money Was: Foreign Bias and English Investment Abroad, 1866–1907’ (2010) 120 Economic Journal 1056.

90 PL Cottrell, *Industrial Finance 1830–1914: The Finance and Organization of English Manufacturing Industry* (Methuen 1980) 270; Michael Dintenfass, *The Decline of Industrial Britain 1870–1990* (Routledge 1992) 41–3; Ranald Michie, *The City of London: Continuity and Change* (Macmillan 1992) 114–16.

91 Janette Rutterford and others, ‘Who Comprised the Nation of Shareholders? Gender and Investment in Great Britain c 1870–1935’ (2011) 64 Economic History Review 157, 161.

92 Michie, *The London Stock Exchange* (n 4) 72; Janette Rutterford and Dimitris P Sotiropoulos, ‘The Rise of the Small Investor in the United States and United Kingdom, 1895 to 1970’ (2017) 18 Enterprise & Society 485, 498 (1.1 million as of 1911).

93 Michie, *The London Stock Exchange* (n 4) 70; Walter Jansson, ‘Stock Markets, Banks and Economic Growth in the UK, 1850–1913’ (2018) 25 Financial History Review 263, 265.

94 Michie, *The London Stock Exchange* (n 4) 143; Jansson (n 93) 265.

95 Michie, *The London Stock Exchange* (n 4) 88–9, 92.

96 ibid 89.

97 Meeghan Rogers, Gareth Campbell and John Turner, ‘From Complementary to Competitive: The London and UK Provincial Stock Markets’ (2020) 80 Journal of Economic History 501, 509.

98 Graeme G Acheson, Gareth Campbell and John D Turner, ‘Active Controllers or Wealthy Rentiers? Large Shareholders in Victorian Public Companies’ (2015) 89 Business History Review 661, 664–5, 676; Leslie Hannah, ‘The Origins, Characteristics and Resilience of the “Anglo-American” Corporate Model’ (2015) 33 Essays in Economic & Business History 1, 11–12; Leslie Hannah, ‘Managerial Capitalism and Corporate Governance’ in Knut Snoger and Andrea Colli (eds), *The Emergence of Corporate Governance: People, Power, and Performance* (Routledge 2021) 10, 11–15, 20–2.

99 Cheffins, *Corporate Ownership and Control* (n 32) 202; Rutterford and others (n 91) 181; Ranald C Michie, ‘The Stock Exchange and the British Economy, 1870–1939’ in van Helten and Cassis (n 86) 95–6. See also Edwards (n 15) 26 (acknowledging the point but saying writers also identified the upside with stock market investments).

100 Cheffins, *Corporate Ownership and Control* (n 32) 197–200.

101 ibid 209, 213–15.

102 ibid 75.

103 ibid 197; Morgan and Thomas (n 15) 153.

104 Morgan and Thomas (n 15) 153.

105 Sturla L Fjesme, Neal E Galpin and Lyndon Moore, ‘Rejected Stock Exchange Applicants’ (2021) 139 Journal of Financial Economics 502; Sturla L Fjesme, Leslie Hannah and Lyndon Moore, ‘Informed Investors, Screening, and Sorting on the London Capital Market, 1891–1913’ Explorations in Economic History (forthcoming).

106 WA Thomas, *The Provincial Stock Exchanges* (Frank Cass 1973) 197–8.

107 Acheson and others (n 75) 932; Acheson, Campbell and Turner, ‘Active Controllers or Wealthy Rentiers?’ (n 98) 668, 683, 690–1.

108 Acheson, Campbell and Turner, ‘Active Controllers or Wealthy Rentiers?’ (n 98).

109 Cheffins, *Corporate Ownership and Control* (n 32) 110–15, 212–13; Gareth Campbell, ‘Substitutes for Legal Protection: Corporate Governance and Dividends in Victorian Britain’ (2011) 64 Economic History Review 571.

110 On World War I developments, see Michie, *The London Stock Exchange* (n 4) 143–5, 148–9, 167–9; Thomas (n 106) 228–9; Chris Swinson, *Regulation of the London Stock Exchange: Share Trading, Fraud and Reform 1914–1945* (Routledge 2018) 84, 86–91.

111 Michie, *The London Stock Exchange* (n 4) 287–8; Thomas (n 106) 239–40; Swinson (n 110) 211–13.

112 H Macmillan, *Report of the Committee on Finance and Industry* (Cmd 3897, 1931) 173 (Macmillan Report).

113 Cheffins, *Corporate Ownership and Control* (n 32) 275; Swinson (n 110) 140.

114 Swinson (n 110) 4, 50, 136–40, 146–50, 166–70.

115 Harold Macmillan, *The Middle Way* (Macmillan 1938) 254, discussing John Maynard Keynes, *The General Theory of Employment Interest and Money* (Macmillan 1936). See also Henry Clay, ‘The Financing of Industrial Enterprise’, Transactions of the Manchester Statistical Society, Session 1931–32, 205, 218 (indicating there had been ‘a good deal of criticism’ of the new issue market).

116 David Chambers, ‘Going Public in Interwar Britain’ (2010) 17 Financial History Review 51, 69.

117 Cheffins, *Corporate Ownership and Control* (n 32) 254, 273–4; Swinson (n 110) 140–1; *Report of the Company Law Amendment Committee* (Cmd 2657, 1926); Companies Act 1928 (18 & 19 Geo 5, c 45), embodied in the Companies Act 1929 (19 & 20 Geo 5, c 23).

118 2 & 3 Geo 6, c 16.

119 Michie, *The London Stock Exchange* (n 4) 292; Heinemann, *Playing the Market* (n 3) 52–8.

120 n 107 and related discussion.

121 Michie, *The London Stock Exchange* (n 4) 258–9; Cheffins, *Corporate Ownership and Control* (n 32) 259, 270.

122 Cheffins, *Corporate Ownership and Control* (n 32) 266; Alan David Chambers, ‘How Well Did the Stock Market Treat Industry? Evidence from Initial Public Offerings on the London Stock Exchange Over the Twentieth Century’ (PhD thesis, London School of Economics 2005) 28–9.

123 Macmillan Report (n 112) 5–6, discussed on this point by Swinson (n 110) 166, 231–2.

124 Macmillan Report (n 112) 173–4, discussed on this point by Michael Collins, *Banks and Industrial Finance in Britain 1800–1939* (Macmillan 1995) 85; David Kynaston, *The City of London, Volume III: Illusions of Gold, 1914–1945* (Chatto & Windus 1999) 227.

125 ‘Money for Small Firms’ *The Times* (London, 22 October 1945); Chris Lonsdale, *The UK Equity Gap: The Failure of Government Policy since 1945* (Ashgate 1997) 37–8, 42; Richard Coopey, ‘The First Venture Capitalist: Financing Development in Britain after 1945, The Case of ICFC/3i’ (1994) 23 Business and Economic History 262, 263–4 (noting as well that the Finance Corporation for Industry was established the same year to focus on larger companies).

126 Above n 5 and related discussion. See also Jeremy Warner, ‘Boardroom Wokery Is Driving Companies out of Public Markets’ *The Telegraph* (London, 22 August 2021); Emma Duncan, ‘The London Stock Exchange Needs Life Support’ *The Times* (London, 5 November 2021); James Ashton, ‘The Stock Market Must Not Be Another Land That Time (and the City) Forgot’ *The Times* (London, 12 December 2022).

127 William Wright, *What Are Stock Exchanges For and Why Should We Care?* (New Financial/Pension Insurance Corporation, 2019) 18.

128 Harold Rose, ‘Industry, New Issues and the Equity Market’ (March 1964) District Bank Review 19, 19.

129 Heinemann, *Playing the Market* (n 3) 66–7, 75; Rutterford and Sotiropoulos (n 92) 521, 525.

130 John Littlewood, *The Stock Market: 50 Years of Capitalism at Work* (Pitman Publishers 1998) 120, focusing on the FT 30 Index, comprised of shares of 30 major industrial firms.

131 Companies Act 1948 (11 & 12 Geo 6, c 38); Companies Act 1967 (c 81). On the relevant changes, see Cheffins, *Corporate Ownership and Control* (n 32) 36, 328–9, 356–8.

132 Cheffins, *Corporate Ownership and Control* (n 32) 36–8.

133 On what was intended, see LCB Gower, *The Principles of Modern Company Law* (3rd edn, Stevens & Sons 1969) 55–6, summarising points raised by *Report of the Committee on Company Law Amendment* (Cmd 6659, 1945); *Report of the Company Law Committee* (Cmnd 1749, 1962).

134 Kieran Heinemann, ‘Popular Investment and Speculation in Britain, 1918–1987’ (PhD thesis, University of Cambridge 2017) 58. See also Heinemann, *Playing the Market* (n 3) 60.

135 On the chronology, see Cheffins, *Corporate Ownership and Control* (n 32) 80–1.

136 Michie, *The London Stock Exchange* (n 4) 358, see also 363; Gower (n 59) 55 (‘many branches of enterprise have already been nationalized’).

137 Rutterford and Sotiropoulos (n 92) 528.

138 ‘An Association of Investors?—(IV) Some Conclusions’ *Financial Times* (3 March 1949).

139 Heinemann, *Playing the Market* (n 3) 60, 129, 131.

140 James Foreman-Peck and Leslie Hannah, ‘Extreme Divorce: The Managerial Revolution in UK Companies before 1914’ (2012) 65 Economic History Review 1217, 1234.

141 Gower (n 59) 289–90.

142 Littlewood (n 130) 250, Table 23.4; Steven Bank, Brian Cheffins and Marc Goergen, ‘Dividends and Politics’ (2004) ECGI Working Paper No 24/2004, Table 6.

143 Cheffins, *Corporate Ownership and Control* (n 32) 342.

144 Finance Act 1965 (c 25), ss 19, 20, 22; ‘Missed Opportunity’ *The Economist* (10 April 1965) 210.

145 ‘City Loyalty “Near Breaking Point”’ *The Telegraph* (London, 31 March 1962).

146 Ivan Fallon, ‘The Last Stand of the Small Investor’ *Sunday Telegraph* (London, 31 July 1977).

147 Committee to Review the Functioning of Financial Institutions, *Evidence on the Financing of Trade and Industry*, vol 3 (HMSO 1979) 198, 208, 212, 218–19. See also ‘Corpse in the Capital Market’ *The Economist* (7 February 1953) 375; Michael Blanden, ‘A Decline of Grass-Roots Capitalism in the UK’ *Financial Times* (26 November 1969) 10.

148 Office for National Statistics, ‘Ownership of UK Quoted Shares 2020’ <www.ons.gov.uk/economy/investmentspensionsandtrusts/bulletins/ownershipofukquotedshares/2020 >.

149 Michie, *The London Stock Exchange* (n 4) 412, 536. The sufficiency of this demand could not be taken for granted: Norman Macrae, *The London Capital Market: Its Structure, Strains and Management* (Staples Press 1955) 175.

150 ‘There is £3½ Billion on the Way, But Plenty to Buy’ *The Economist* (1 March 1975) 84.

151 Cheffins, *Corporate Ownership and Control* (n 32) 346–52.

152 Littlewood (n 130) 107–8, 122.

153 Office for National Statistics (n 148).

154 Brian R Cheffins, *The Public Company Transformed* (OUP 2019) 227.

155 See eg Coyle, Musacchio and Turner (n 16) 280; Leslie Hannah, ‘The London Stock Exchange, 1869–1929: New Statistics for Old?’ (2018) 71 Economic History Review 1349.

156 Raghuram G Rajan and Luigi Zingales, ‘The Great Reversals: the Politics of Financial Development in the Twentieth Century ‘(2003) 69 Journal of Financial Economics 5, 15 (0.38 in 1980); Rafael La Porta, Florencio Lopez-de-Silanes and Andrei Shleifer, ‘Law and Finance after a Decade of Research’ in George M Constantinides, Milton Harris and Rene M Stulz (eds), *Handbook of the Economics of Finance*, vol 2A (North Holland, 2013) 425, 475 (0.76 in 1977); World Bank, ‘Market Capitalization of Listed Domestic Companies (% of GDP)—United Kingdom’ <https://data.worldbank.org/indicator/CM.MKT.LCAP.GD.ZS?locations=GB> (0.06 in 1980).

157 Hannah, ‘Managerial Capitalism’ (n 98) 14, Table 1.1; La Porta, Lopez-de-Silanes and Shleifer (n 156) 474; Rajan and Zingales (n 156) 15.

158 Cheffins and Reddy (n 8) 180, Figure 2; World Bank (n 156).

159 n 128 and related discussion; London Stock Exchange Reports <www.londonstockexchange.com/reports?tab=issuers>; London Stock Exchange, *Fact Book: 1993* (London Stock Exchange 1993).

160 James J Fishman, *The Transformation of Threadneedle Street: The Deregulation and Reregulation of Britain’s Financial Services* (Carolina Academic Press 1993) 244–5.

161 Richard Wachman, ‘Decline and Fall of the Cult of Shares’ *The Observer* (London, 6 July 2008). See also John Littlewood, *The Stock Market under Labour* (Centre for Policy Studies 2004) Appendix 2 (share prices increased 167% between 1979 and 1997, adjusted for inflation); *The Kay Review of UK Equity Markets and Long-Term Decision-Making: Final Report* (Department for Business, Innovation and Skills 2012) 18 (1982–2000 data).

162 World Bank (n 156) (reporting a steady increase from 0.06:1 in 1980 to 1.75:1 in 1999).

163 Hannah, ‘London as the Global Market’ (n 14) 149.

164 Gerald Colverd, ‘Can Small Investors Succeed?’ *Sunday Telegraph* (London, 22 May 1983).

165 ibid; Heinemann, *Playing the Market* (n 3) 156; Clive Wolman, ‘Wider Share Ownership’ *Financial Times* (11 January 1986).

166 John Edwards, ‘Wider Share Ownership’ *Financial Times* (17 January 1987).

167 Heinemann, *Playing the Market* (n 3) 191, 194; Michie, *The London Stock Exchange* (n 4) 491, 543–54, 559–60, 588; Fishman (n 160) 33–5, 244–5.

168 Michie, *The London Stock Exchange* (n 4) 501–2, 578.

169 ‘The USM Has Arrived’, *The Times* (London, 7 November 1980).

170 John H Farrar, Nigel E Furey and Brenda M Hannigan, *Company Law* (3rd edn, Butterworths 1991) 549–50; *Report of the Committee to Review the Functioning of Financial Institutions (Harold Wilson, chair)* (Cmnd 7937, 1980) 214–16, 313–14.

171 Anthony Hilton, ‘Looser Exchange Rules Mean USM Is Redundant’ *Accountancy Age* (3 March 1994) 13.

172 Edwards (n 15) 65 (‘immediate success’); McRae and Cairncross (n 60) 154 (‘considerable success’); Colverd (n 164) (‘great success’).

173 David Kynaston, *The City of London, Vol IV: A Club No More, 1945–2000* (Chatto & Windus 2001) 618.

174 John Burton, ‘Privatization: The Thatcher Case’ (1987) 8 Managerial and Decision Economics 21, 24; Matthew R Bishop and John A Kay, ‘Privatization in the United Kingdom: Lessons from Experience’ (1989) 17 World Development 643, 647; Chris Edwards, ‘Margaret Thatcher’s Privatization Legacy’ (2017) 37 Cato Journal 89, 89, 91–2.

175 Alistair Osborne, ‘Privatisation: The Trademark Policy That Was Copied Round the World’ *The Telegraph* (London 9 April 2013).

176 Mike Toms, ‘The Thatcher Privatisation Legacy: Not Quite What She Planned?’ (*Oxera Agenda*, May 2013) 1.

177 Edwards (n 174) 98.

178 Burton (n 174) 24, 26; Bishop and Kay (n 174) 649; Robert Ansted, ‘Thatcher’s Legacy’ *Investors’ Chronicle* (26 July 2013) <www.investorschronicle.co.uk/2013/07/25/shares/news-and-analysis/thatcher-s-legacy-QmrUh1kUPtkP9Q4YlVqX3L/article.html>.

179 Burton (n 174) 24–5; Toms (n 176); Ansted (n 178); John Moore, ‘Privatization: Taking Capitalism to the People’ [January/February 1992] Harvard Business Review 115, 118–19.

180 Janet Bush, ‘How the Conservatives Have Won Sid’s Vote’ *Financial Times* (2 June 1987) 10; Jonathan Guthrie, ‘How the BT-Led Revolution Fizzled Out’ *Financial Times* (4 December 2004) Money and Business, 3; John Redwood, ‘Thatcher Allowed More People to Participate in the Wealth of the Nation through Property Ownership and Shares’ *The House* (3 May 2019) <www.politicshome.com/thehouse/article/thatcher-allowed-more-people-to-participate-in-the-wealth-of-the-nation-through-property-ownership-and-shares >.

181 ‘Breadth without Depth’ *Financial Times* (31 March 1987).

182 Bush (n 180) (advertising); Terry Dodsworth, ‘Investors Take the Money and Run’ *Financial Times* (16 December 1989); Roger Buckland, ‘The Costs and Returns of the Privatization of Nationalized Industries’ (1987) 65 Public Administration 241, 253–4 (generous terms); McRae and Cairncross (n 60) 156.

183 Matthew Vincent, ‘25 Years On …What Could Sid Tell Us?’ *Financial Times* (10 December 2011).

184 Edwards (n 166).

185 Brian Groom and Sylvia Pfeifer, ‘Privatising Zeal That Defined Thatcher Era Lives on 25 Years Later’ *Financial Times* (8 December 2011). See also Vincent (n 183) (privatisation ‘failed to usher in a new era of popular share ownership’); Nick Gardner, ‘Floats Take Sid Out of His Depth’ *Sunday Times* (London, 21 July 1996) (the plan to foster ‘a whole-hearted share-owning philosophy … clearly failed’).

186 ‘The Private Shareholder’ *Financial Times* (18 October 1990); Gillian O’Connor, ‘So Long Sid, Goodbye Maud’ *Financial Times* (10 February 1996); Matthew Wall, ‘Shares for All: The Vision That Faded’ *Financial Times* (22 February 1997).

187 Heinemann, *Playing the Market* (n 3) 153, 226; Dodsworth (n 182); ‘Risk Aversion’, *The Economist* (6 November 1993) 79.

188 Groom and Pfeifer (n185); Tanya Jeffries, ‘What Happened to Thatcher’s Share Ownership Dream?’ *ThisMoney* (9 October 2015) <www.thisismoney.co.uk/money/investing/article-3265284/What-happened-Margaret-Thatcher-s-share-ownership-dream.html>.

189 Gardner (n 185); Wall (n 186).

190 Office for National Statistics (n 148).

191 ibid; Edwards (n 15) 7.

192 Edwards (n 174) 94, Table 1; Guthrie (n 180); Vincent (n 183).

193 Maggie Urry, ‘Stock Exchange Recommends Closure of USM by end-1995’ *Financial Times* (22 December 1992). See also ‘Unloved Toddlers’ *The Economist* (6 March 1993) 102 (the USM ‘was dying on its feet’); Philip Whiterow, ‘Re-inventing the Smaller Companies Wheel’ *Investors Chronicle* (24 September 1993) 16 (the USM’s ‘usefulness had passed’). On the 1980s angle, see n 173 and related discussion; Hamish McRae, ‘Learning from the USM’s Demise’ *The Independent* (London 22 December 1992); Anthony Hilton, ‘Replace the USM with a Professional’s Market’ *Accountancy Age* (22 July 1993) 11.

194 Ronald Cohen, ‘Special Care for Young Companies’ *Financial Times* (8 March 1994). See also Michie, *The London Stock Exchange* (n 4) 619; Sridhar Arcot, Julia Black and Geoffrey Owen, *From Local to Global: The Rise of AIM as a Stock Market for Growing Companies* (LSE 2007) 12 (data on the amount of new capital raised showing the same pattern).

195 Hilton (n 193); Arcot, Black and Owen (n 194) 12. On the nature of EU capital market regulation affecting the UK during this era, see Eilis Ferran and Look Chan Ho, *Principles of Corporate Finance Law* (2nd edn, OUP 2014) 360–1.

196 McRae (n 193); Urry (n 193).

197 ‘Unloved Toddlers’ (n 193).

198 Urry (n 193); Arcot, Black and Owen (n 194) 12. For a defence of the LSE’s stance, see Andrew Hugh Smith, ‘Two May Be a Crowd’ *Financial Times* (8 March 1994) 20.

199 Arcot, Black and Owen (n 194) 12. On private sector lobbying, see also Whiterow (n 193); Richard Balarkas, ‘The Cisco Kid’ [September 1993] APCIMS Newsletter 9; Richard Gourlay, ‘Secondary Market with Big League Rules’ *Financial Times* (16 February 1995). On government pressure, see Neil Thapar, ‘Government Urges Successor to USM’ *Independent on Sunday* (London 27 March 1994); ‘Small Companies’ *Financial Times* (16 February 1995).

200 Gourlay (n 199).

201 David Hellier, ‘AIM Meets the Deadline for Its Debut’ *The Independent* (London, 14 June 1995).

202 Lex, ‘Aim’ *Financial Times* (19 June 1996).

203 Sarah Whitebloom, ‘AIM Proves It Is on Target’ *The Observer* (London, 5 May 1996).

204 Kevin Root, ‘Aiming at Risk and Reward’ *The Telegraph* (London, 6 April 1996); Claire Oldfield, ‘Bullish Outlook for £3.4bn AIM’ *Sunday Times* (London, 16 June 1996).

205 Arcot, Black and Owen (n 194) 9.

206 SJ Gray and MC McDermott, *Mega-merger Mayhem: Takeover Strategies, Battles and Controls* (Sage Publications 1989).

207 For an overview of this line of thinking during the 1980s, see Clive Wolman, ‘Keynes Followers Rally behind Industrialists’ *Financial Times* (3 March 1987); David Goodhart and Charles Grant, *Making the City Work* (Fabian Society 1988) 17–19.

208 ‘At Odds, Again’ *The Economist* (2 March 1991) 94; David Clutterbuck and David Bowen, *The Decline and Rise of British Industry* (Mercury Books 1988) 311–14; David Bowen, *Shaking the Iron Universe: British Industry in the 1980s* (Hodder & Stoughton 1990) 302 (discussing a 1985 speech by David Walker, a director of the Bank of England).

209 Philip Stephens and Geoffrey Owen, ‘Short Termers, Short Shrift’ *Financial Times* (5 January 1987).

210 Paul Marsh, *Short Termism on Trial* (IFMA 1990) 4.

211 ‘The Battle for Dividends’ *Financial Times* (1 June 1994) 23; Ronald Rudd, ‘Hanson Angered by Dorrell Query over “Too High” Dividends’ *Financial Times* (4 June 1994) 1; Chris Lonsdale, *The UK Equity Gap: The Failure of Government Policy since 1945* (Ashgate 1997) 34–5.

212 John Kingman, ‘Audacity? That’s Only the Half of It’ *Financial Times* (5 July 1997); Roni Michaely and Michael R Roberts, ‘Corporate Dividend Policies: Lessons from Private Firms’ (2012) 35 Review of Financial Studies 711, 738.

213 Kingman (n 212); ‘Investment Brown-Out’ *The Economist* (5 July 1997) 23.

214 On other factors, see Cheffins, *Corporate Ownership and Control* (n 32) 389–90.

215 Chris Brown-Humes, ‘UK Market Has De-equitisation to Thank for Post-2004 Form’ *Financial Times* (26 August 2006).

216 Cheffins, *Corporate Ownership and Control* (n 32) 387–8.

217 Office for National Statistics (n 148).

218 Tony Tassel and Lina Saigol, ‘International Investors in the UK Are Buying Up the Keys to the Kingdom’ *Financial Times* (22 June 2005).

219 Pauline Skypala, ‘Sacrificed to the Winds of Change’ *Financial Times* (1 March 2010); John Plender, ‘The Pensions Shake-Up Heralds Equity Demand Change’ *Financial Times* (20 March 2010).

220 Anthony Hilton, ‘Falling Out of Love with Shares’ *Evening Standard* (London, 10 June 2004).

221 Cheffins and Reddy (n 8) 180–1.

222 ibid 179, Figure 1.

223 John A Doukas and Hafiz Hoque, ‘Why Firms Favour the AIM When They Can List on Main Market?’ (2016) 60 Journal of International Money and Finance 378, 384.

224 Tim Howard, ‘Is Aim in the Last-Chance Saloon or Is Junior Market Set to Revive?’ *The Times* (London, 28 October 2019) 42. For annual data on companies listed on AIM, see Quoted Companies Alliance, ‘Call for Evidence—UK Listings Review’ (5 January 2021) Appendix 2, <www.theqca.com/news/responses/229011/qca-response-to-the-uk-listings-review.thtml>.

225 Cheffins and Reddy (n 8) 179, Figure 1.

226 ibid Figure 2.

227 Hilton (n 220).

228 ‘Long-Termism and the Stock Market’ *Financial Times* (24 July 2012).

229 Paul Davies, *Gower and Davies Principles of Modern Company Law* (9th edn, Sweet & Maxwell 2012) 17, 902.

230 Chris Hughes and Joanna Chung, ‘City Troubled by Foreign Companies’ *Financial Times* (4 November 2006) 17; Brian R Cheffins, ‘The Undermining of UK Corporate Governance (?)’ (2013) 33 OJLS 503, 505–6.

231 Cheffins, ‘The Undermining of UK Corporate Governance’ (n 230) 513–14; Isabel Gorst et al, ‘The Eurasian Equation’ *Financial Times* (24 June 2011); Jonathan Guthrie, David Oakley and Lina Saigol, ‘New Money Puts City’s Reputation at Risk’ *Financial Times* (18 May 2013).

232 Cheffins, ‘The Undermining of UK Corporate Governance’ (n 230) 511–13; ‘Mining Accidents’ *Financial Times* (24 April 2013).

233 Jim Pickard and Kate Burgess, ‘Cable Launches Stock Market Review’ *Financial Times* (22 June 2011).

234 ‘Vince Cable’s Speech to Lib-Dem Conference Decoded by Angela Monaghan’ *The Telegraph* (London, 23 September 2010).

235 John Kay, ‘Don’t Tell Sid but It’s All Monopoly Money’ *The Telegraph* (10 December 1986).

236 John Kay, ‘Corporate Strategy and Corporate Accountability’ in Nicholas Dimsdale and Martha Prevezer (eds), *Capital Markets and Corporate Governance* (OUP 1994) 50–1, 59–63; John Kay, *The Business of Economics* (OUP 1996) 108–21.

237 ‘Professor Kay’s Speech: Call for Evidence Launch’ (15 September 2011) 9 <https://assets.publishing.service.gov.uk/government/uploads/system/uploads/attachment_data/file/31546/kay-review-napf-speech-sept-2011.pdf>.

238
*Kay Review* (n 161) 24–5.

239 ibid 62.

240 ibid 18–21.

241 ibid 22–4.

242 ibid 25.

243 ibid 62.

244 ibid 82.

245 ibid 9.

246 Department for Business, Innovation and Skills, ‘Ensuring Equity Markets Support Long-Term Growth—The Government Response to the Kay Review’ (November 2012) 15–17 <www.gov.uk/government/uploads/system/uploads/attachment_data/file/253457/bis-12-1188-equity-markets-support-growth-response-to-kay-review.pdf >.

247 HC Deb 9 November 2020, vol 683, col 619 <https://hansard.parliament.uk/commons/2020-11-09/debates/D5E911A9-1270-457F-9F6A-57AE5C272FBA/FutureOfFinancialServices>.

248 FCA, Listing Rules, 5.2.2(2)G, 6.14.2(2)R, 14.2.2(3)R (free float), 5.6.8G, 5.6.18AG–FG (SPACs); 9.2.22AR–FR (voting rights).

249 FCA (n 9).

250 Ian King, ‘Why Are Listed Company Bosses Asking: “Is This Worth the Hassle?”’ *The Times* (London, 28 May 2019); Jeremy Warner, ‘Only a Revolution Will Revitalise Our Unloved Public Markets’ *The Telegraph* (London, 1 January 2020); Quoted Companies Alliance and Peel Hunt, ‘New Research Shows UK Public Markets in Decline, as Listed-Life Loses Appeal’ (27 January 2020) <www3.theqca.com/news/press-releases/197516/press-releasenew-research-shows-uk-public-markets-in-decline-as-listedlife-loses-appeal.thtml.>

251 ‘Brex and the City’ *The Economist* (24 October 2020) 23.

252 Quoted Companies Alliance and Peel Hunt (n 250).

253 Augar (n 11).

254 ‘Brex and the City’ (n 251).

255 HM Treasury, ‘Call for Evidence—UK Listing Review’ (19 November 2020) <www.gov.uk/government/publications/uk-listings-review/call-for-evidence-uk-listings-review>.

256 For example, the FCA has proposed deregulating rules governing related party transactions, a form of protection which law and finance experts have identified as crucial for fostering stock market development: FCA (n 9) 46–52, fnn 22, 27 and accompanying text.

257 ‘Bourse Correction’ *The Economist* (6 May 2023) 19. For an overview of the potential causes of the decline of UK equity markets, see Brian R Cheffins and Bobby V Reddy, ‘Murder on the City Express—Who Is Killing the London Stock Exchange’s Equity Market?’ (2023) 44 Co Law 215.

258 FCA (n 9) 3.

